# RETRACTION: Prevalence of Wound Infections and Postoperative Complications after Total Elbow Arthroplasty for Rheumatoid Arthritis: A Meta‐Analysis

**DOI:** 10.1111/iwj.70325

**Published:** 2025-03-06

## Abstract

**RETRACTION:** ChenQ.‐Y. , LiuL. , and GuF.‐Z. , “Prevalence of Wound Infections and Postoperative Complications after Total Elbow Arthroplasty for Rheumatoid Arthritis: A Meta‐Analysis,” International Wound Journal 21, no. 2 (2024): e14451, 10.1111/iwj.14451.PMC1082812037867410

The above article, published online on 22 October 2023, in Wiley Online Library (http://onlinelibrary.wiley.com/), has been retracted by agreement between the journal Editor in Chief, Professor Keith Harding; and John Wiley & Sons Ltd. Following an investigation by the publisher, all parties have concluded that this article was accepted solely on the basis of a compromised peer review process. The editors have therefore decided to retract the article. The authors did not respond to our notice regarding the retraction.

**RETRACTION:** Q.‐Y. Chen , L. Liu , and F.‐Z. Gu , “Prevalence of Wound Infections and Postoperative Complications after Total Elbow Arthroplasty for Rheumatoid Arthritis: A Meta‐Analysis,” International Wound Journal 21, no. 2 (2024): e14451, 10.1111/iwj.14451.PMC1082812037867410

The above article, published online on 22 October 2023, in Wiley Online Library (http://onlinelibrary.wiley.com/), has been retracted by agreement between the journal Editor in Chief, Professor Keith Harding; and John Wiley & Sons Ltd. Following an investigation by the publisher, all parties have concluded that this article was accepted solely on the basis of a compromised peer review process. The editors have therefore decided to retract the article. The authors did not respond to our notice regarding the retraction.

